# Human genetic variation influences *Plasmodium falciparum* drug resistance selection

**DOI:** 10.1186/1475-2875-13-S1-P66

**Published:** 2014-09-22

**Authors:** Giacomo Maria Paganotti, Baba Christiane Gallo, Federica Verra, Bienvenu Sodiomon Sirima, Issa Nebie’, Amidou Diarra, Mario Coluzzi, David Modiano

**Affiliations:** 1 University of Botswana, University of Pennsylvania Partnership, Gaborone, Botswana; 2 University La Sapienza, Rome, Italy; 3 Centre National de Recherche et Formation sur le Paludisme, Ouagadougou, Burkina Faso

## 

Here we address the issue of the possible interplay between host genetic variation and the risk of acquiring *Plasmodium falciparum* drug-resistant strains. The involvement of human genetic variation as a possible co-factor in the selection and spread of *P. falciparum* drug resistance is a new tool in the study of malaria and possibly of other infectious diseases. The driving hypothesis of this approach is that parasite drug resistance could be affected both by ethnicity and human variability in the genes encoding for enzymes that metabolise antimalarials (cytochrome P450 liver enzymes). Understanding if parasite drug sensitivity is influenced and possibly modulated by human diversity can contribute to a better knowledge and control of the spread of drug resistance. So far, few studies have addressed this strategic issue. To explore this hypothesis we carried out an association analysis on 506 human/*P. falciparum* DNA samples from adult asymptomatic subjects belonging to three sympatric ethnic groups of Burkina Faso, an area of hyperendemic malaria in West Africa. Here we report that the prevalence of chloroquine-resistant infections (*pfcrt* 76T and/or *pfmdr1* 86Y) differs among sympatric ethnic groups, being higher in Mossi and Rimaibé compared to Fulani (OR: 2.24; 1.27-3.92; *P* = 0.007). Moreover, the human CYP2C8*2 variant, known to determine a poor drug metaboliser phenotype, is associated with *P. falciparum* chloroquine-resistant infections (OR: 1.66; 1.13-2.43; *P* = 0.008). The results strongly suggest that human genetic variation affects the dynamics of selection of parasite drug-resistance. We strongly believe that these observations are of general interest and may have important implications in public health.

